# Use of the new Lake Louise Criteria improves CMR detection of atypical forms of acute myocarditis

**DOI:** 10.1007/s10554-020-02097-9

**Published:** 2020-11-15

**Authors:** Giulia Cundari, Nicola Galea, Gianluca De Rubeis, Andrea Frustaci, Francesco Cilia, Giuseppe Mancuso, Livia Marchitelli, Federica Catapano, Iacopo Carbone, Carlo Catalano, Marco Francone

**Affiliations:** Department of Radiological, Oncological and Anatomopathological Sciences, Sapienza University of Rome, Rome, Italy

**Keywords:** Myocarditis, Agreement, Diagnostic accuracy, Lake Louise criteria, Atypical onsets

## Abstract

The purpose of our study was to compare diagnostic performance of old and new Lake Louise Criteria (oLLC and nLLC) among different clinical presentations: infarct-like (IL), cardiomyopathic (CM) and arrhythmic (AR). 102 patients with clinical suspicion of acute myocarditis underwent cardiac magnetic resonance (CMR) on a 1.5 T scanner. Protocol included cine-SSFP, T2-weighted STIR, T2 mapping, early and late gadolinium enhancement and T1 mapping acquired before and after gadolinium administration. The degree of agreement has been calculated with Cohen’s K test. 42 patients also underwent endomyocardial biopsy (EMB). IL onset was present in 54/102 patients, CM in 28/102 and AR in 20/102. nLLC were positive in 58.3% of the patients, while oLLC in 37.9%, k = 0.57 (IC: 0.428–0.713). The degree of agreement between nLLC and oLLC was 0.49 (IC: 0.111–0.876) for AR onset (nLLC positive in 35% vs oLLC in 15%), 0.25 (IC: 0.035–0.459) for CM pattern (nLLC positive in 60.7% vs oLLC 17.9%) and 0.73 (IC: 0.543–0.912) for IL presentation (nLLC positive in 66.7% vs oLLC in 57.4%). Diagnostic accuracy was 75% for both nLLC and oLLC among IL onset, and 41.6% for oLLC vs 66.7% for nLLC, as regards CM clinical presentation. nLLC have improved diagnostic performance of CMR for the diagnosis of acute myocarditis, in particular for atypical clinical presentation.

## Introduction

Clinical presentations of acute myocarditis (AM) vary through a wide range of manifestations [1, 2], affecting either children [3] or adults [4]. The infarct-like onset, the most common clinical pattern [5] which seems to be mostly correlated to Parvovirus B19 infection [6], is characterized by flu-like symptoms followed by chest pain, typical electrocardiogram (ECG) features, myocardial necrosis biomarkers release (in particular T-troponin) and normal coronary angiogram [7]. In other patients, myocarditis shows a cardiomyopathic presentation with acute or chronic heart failure, systolic impairment with or without left ventricle dilation or with new onset ventricular arrhythmias, high-degree atrio-ventricular block or bundle branch block [2, 8]. Other confounding features are the variable clinical course (myocarditis can be acute, subacute, or chronic [9]), the underlying pathological substrate and the aetiology of the disease (viral or bacterial infections [10], toxins or drugs or autoimmune disorders [10–12]).

Endomyocardial biopsy (EMB) still represents the gold standard for the diagnosis of acute myocarditis [13], that is based on the Dallas criteria [14], which have been improved by immunohistochemical analysis and Polymerase Chain Reaction (PCR) [15]. However, EMB is an invasive procedure with several limits, such as the lack of standard protocols, sampling errors and the low sensitivity [16–18].

In 2009, Friedrich et al. defined the Consensus Criteria for CMR in myocardial inflammation, the so-called Lake Louise Criteria (LLC). According to LLC, AM diagnosis was established with the presence of at least 2 out of 3 of the following CMR features: oedema (with T2-weighted sequences), hyperaemia (with Early Gadolinium Enhancement–EGE) and necrosis or fibrosis (with Late Gadolinium Enhancement–LGE) [19].

New LLC (nLLC) were recently published following the introduction of myocardial mapping and redefined imaging diagnosis according to the combined presence of a T1 criterion (presence of LGE or increased T1 mapping or extracellular volume values) and a T2 criterion (hyperintensity in T2 weighted STIR or increased T2 mapping values) [20].

According to the literature, the sensitivity of old Lake Louise Criteria (oLLC) varied with different clinical presentations (ranging from an 80% for the infarct-like onset to a 40% for the arrhythmogenic pattern [21]).

To the best of our knowledge, the clinical performance of nLLC has not been tested yet in relation to different clinical presentations and in particular for atypical onsets. Our hypothesis was that parametric imaging could recognize subtle or diffuse forms of the disease, by quantitatively measuring T1 and T2 relaxation time variations.

The purpose of our study was therefore to compare the diagnostic performance of old and new LLC among the different clinical patterns: infarct-like (IL), cardiomyopathic (CM) and arrhythmic (AR).

## Materials and methods

### Study population

We retrospectively analysed 102 patients referred to our institution for suspected acute myocarditis. Exclusion criteria were defined as follows: evidence of coronary artery disease, valvular diseases or cardiomyopathies; systemic pathologies; pacemaker or implantable cardiac defibrillator; renal insufficiency or risk of nephrogenic systemic fibrosis (glomerular filtration < 30 ml/min/1.73 m^2^); contrast media allergies; pregnancy or nursing; clinical history > 30 days [11]. 42 (42/102, 41.1%) also underwent EMB.

Patient’s categorization was based on well-defined clinical features, as previously addressed by our group [21]:

- Those who presented with chest pain, increase in T-Troponin serum level (≥ 0.014 μg/ml) and ST-T tract elevation were grouped in the IL onset
- Patients with systolic impairment (ejection fraction < 54%), left ventricle (LV) dilation and heart failure symptoms in the absence of ECG ST-T abnormalities were categorized as CM;
- Subjects with new onset arrhythmias (atrial fibrillation, ventricular arrhythmias, high-degree atrio-ventricular block or bundle branch block) with or without heart failure symptoms were grouped in the AR pattern.

The categorization of clinical presentations was performed by a blinded radiologist at the time of the database preparation. Local ethic committee approved our study.

### CMR protocol

After having obtained written informed consent, CMR was performed on a 1.5 T scanner (Magnetom Avanto, Siemens medical systems, Erlangen, Germany), using body and phased array coils.

A single dose of 0.25 ml/kg of gadoteric acid (Claricyclic, GE healthcare, Chicago, Illinois, USA) was injected intravenously at a flow rate of 2 ml/s.

Protocol included cine steady-state free precession (cine-SSFP) acquired during breath-holds in short-axis (from the base to the apex, 10–12 slices), 2-chamber, and 4-chamber planes (TR: 51.3 ms, TE: 1.21 ms, flip angle: 45°, slice thickness: 8 mm, matrix: 256 × 256, field of view: 340–400 mm, voxel size: 2.0 × 1.3 × 8.0 mm). T2-weighted breath-hold black-blood STIR were obtained using a segmented turbo spin echo triple inversion recovery technique on short axis (from the base to the cardiac apex, 8–10 slices), 2 chambers and 4 chambers planes (TR: 2 R–R intervals, TE: 75 ms, flip angle 180°, TI: 170 ms, slice thickness: 8 mm, Field of view: 340–400 mm, matrix: 256 × 256, voxel size: 2.3 × 1.3 × 8 mm). T1-weighted turbo spin echo (TSE) images were acquired with a non–electrocardiogram (ECG)-gated sequence before and immediately after contrast media administration on the same 4–5 axial slices.

A phase sensitive inversion recovery gradient echo (PSIR-GRE) contrast-enhanced sequence was used 10–15 min after gadolinium injection for the LGE (TI: 250–300 ms, TR: 9.6 ms; TE: 4.4 ms; matrix: 256 × 208; flip angle 25°; slice thickness 5.0 mm; slice spacing 5.0 mm).

For T1-mapping, we used a Modified Look Locker Inversion Recovery (MOLLI) sequence, with a 5(3)3 scheme. MOLLI were acquired in three axial slices (basal, mid-ventricular and apical planes) and in four-chambers view, before contrast agent administration and 15 min after Gadolinium injection (TR: 314 ms; TE: 1.12 ms; flip angle: 35°; TI: 200 ms; slice thickness: 8 mm; field of view: 340–400 mm; matrix: 256 × 256; voxel size: 2.1 × 1.4 × 8 mm).

T2 mapping was acquired with a T2-3pt GRE in short axis through basal, mid ventricular and apical planes (TR: 239 ms; TE: 1.13 ms; Flip angle: 12°; Slice thickness: 8 mm; field of view 340–400 mm; matrix 256 × 256; voxel size: 2.5 × 1.9 × 8 mm).

### Images analysis

Images were analysed in blind using a dedicated software (Cvi42, Calgary, Canada) by two radiologists with 2 years and 10 years of experience, respectively.

Global and regional ventricular function were assessed using cine-SSFP. Endocardial and epicardial contours were traced on end-diastolic and end-systolic images acquired in short axis planes, in order to determine left ventricular volumes and ejection fraction (EF).

The presence of oedema was evaluated both with T2w-STIR sequences and with T2 mapping. As regards STIR images, a visual assessment of oedema was performed with a signal intensity (SI) > 2 standard deviation (SD) above the remote myocardium. For the semi-quantitative analysis, we used the T2 ratio technique in order to relate the SI of the myocardium to that of the skeletal muscle. We outlined endocardial and epicardial contours in the short axis planes and we drew a Region of Interest (ROI) within a visible skeletal muscle. A ratio ≥ 2 was considered positive for oedema [19]. For T2 mapping sequences, we manually traced endocardial and epicardial contours throughout basal, mid and apical slice, and positioned a reference point in the anterior interventricular junction. Thanks to our dedicated software, we were able to identify T2 values for each AHA segment [22]. Then, we calculated mean slice T2 values oedema for each plane. We considered the presence of oedema for mean slice T2 values of more than 50 ms.

Hyperaemia was evaluated with T1-TSE sequences. We traced endocardial and epicardial contours in axial slices acquired before gadolinium injection and then copied contours in slices acquired immediately after contrast media administration. We also drew a ROI within a visible skeletal muscle in order to calculate EGE ratio (EGEr). Hyperaemia was considered to be positive with an EGEr ≥ 4 or with an absolute increase in myocardial SI of 45% in the contrast-enhanced T1-TSE as compared to the pre-contrast images [20].

As regards necrosis/fibrosis, LGE was identified with a signal intensity > 5 SD [23] above the remote myocardium and a non-ischemic pattern of distribution (subepicardial or mesocardial or patchy enhancement) [19].

T1 mapping analysis was performed tracing endocardial and epicardial contours on the basal, mid and apical slices acquired before and 15 min after Gadolinium injection. A ROI was drawn within the LV cavity in order to obtain T1 value of the blood pool. Finally, a reference point was positioned in the anterior interventricular junction.

Our software identified native T1 values and calculated extracellular volume (ECV) for each AHA segment [22] according to the formula described elsewhere [24]. Haematocrit value was acquired within 24 h before the scan.

Native T1 mapping was considered to be pathological with mean slice T1 values of more than 1050 ms; ECV was identified as pathological with a mean slice percentage ≥ 28%.

### Endomyocardial biopsy

42 (42/102, 41.1%) patients also underwent EMB, in accordance with the American Heart Association, the American College of Cardiology and the European Society of Cardiology recommendations [16]. EMB procedure performed in our Institution is explained elsewhere [21].

### Statistical analysis

Statistical analysis was performed using Medcalc software Ltd (Ostend, Belgium).

The clinical and demographic features were compared using a two-way Chi-Squared Test. Kruskal–Wallis test was used for comparing ventricular functional parameters between the three population (IL, CM and AR).

Inter-observer agreement and the level of agreement between old and new LLC were assessed with Cohen’s k test. Agreement based on *k* values was interpreted as follows: below 0.4 as poor, between 0.41 and 0.60 as moderate agreement, between 0.61 and 0.80 as substantial agreement, and between 0.81 and 1 as almost perfect. McNemar’s test (MNT) was used to compare the diagnostic proportion between oLLC and nLLC among different clinical presentations. Diagnostic accuracy was calculated only in the selected subcohort of patients who underwent EMB, as the proportion between true positive and true negative and is expressed as a percentage followed by Clopped-Pearson Confidence Interval.

The net reclassification improvement (NRI) and the net absolute reclassification improvement (NARI) were also calculated.

A p-value < 0.05 was considered statistically significant.

## Results

### Left ventricular volumes and function

Mean population age was 44.8 ± 17.2 years, females were 35 (34.3%).

The average time between the onset of symptoms and CMR was 6 ± 1.7 days, with a range of 3–20 days following ER admission.

54 (54/102, 52.9%) patients presented with IL onset, 28 (28/102, 27.5%) with CM and 20 (20/102, 19.6%) with AR. Within the IL forms, three individuals presented with severe heart failure symptoms associated with remarkable T-troponin release and ST-T abnormalities, and were categorized as “fulminant myocarditis”. In one of those patients, ventricular mechanical support was needed, due to the occurrence of cardiogenic shock.

For ventricular function, we found statistically significant differences between CM group compared to IL and AR as regards end diastolic volume (EDV) normalized to Body Surface Area (BSA) and EF. EDV/BSA and EF are expressed as median and confidence interval (CI). EDV/BSA was 109.75 [89–121.15] ml/m^2^ for CM vs 76.2 [74.3–83.5] ml/m^2^ for IL and 76.1 [67.65–80.85] ml/m^2^ for AR, p < 0.05. EF was 33.9% [27.02–43.99%] for CM vs 56.04% [54.38–58.87%] for IL and 55.2% [49.78–60.10%] for AR, p < 0.05 (see Table 1).

**Table 1:** The table summarizes main population clinical features, CMR functional parameters, median native T1, T2 mapping and ECV values both for the suspected myocarditis and for the biopsy-proven positive cases, the level of agreement between oLLC and nLLC, endomyocardial biopsy histological diagnosis and the diagnostic accuracy of oLLC and nLLC in relation to different clinical presentations (IL and CM)

		IL	CM	AR	p-value
Onset clinical features	Patients population	54/102, 52.9%	28/102, 27.5%	20/102, 19.6%	< 0.05
	Age	41.3% ± 18.1	48.5 ± 16.3	47.8 ± 17.3	> 0.05
	Sex	16/54, 29.6% F	9/28, 32.1% F	10/20, 50% F	< 0.05
	Fever or flu like symptoms (W)	53/54, 98.1%	15/28, 53.6%	2/20, 10%	< 0.05
	Dyspnoea (W)	25/54, 46.3%	20/28, 71.4%	5/20, 25%	< 0.05
	Chest pain (W)	54/54, 100%	3/28, 10.7%	7/20, 35%	< 0.05
	Troponine T ≥ 0.014 (gL) (W)	50/54, 92.6%	0/28	0/20	< 0.05
	ECG ST-T abnormalities (W)	49/54, 90.7%	0/28	1/20, 5%	< 0.05
	Arrhythmias (No)	3/54, 5.56%	10/28, 35.7%	20/20, 100%	< 0.05
CMR parameters	EDV/BSA (ml/m^2^, median and CI)	76.2 [74.3–83.5]	109.75 [89–121.15]	76.1 [67.65–80.85]	< 0.05
	EF (%, median and CI)	56.04 [54.38–58.87]	33.9 [27.02–43.99]	55.2 [49.78–60.10]	< 0.05
	nTI (ms, median and CI)	1057 [1042–1077]	1093 [1080–1133]	1029 [1008–1054]	< 0.05
	T2 (ms, median and CI)	48.75 [48.90–50.92]	51.36 [50.53–54.33]	49.83 [48.85–51.99]	< 0.05
	ECV (%, median and CI)	26.74 [26.82–29.20]	30.18 [27.37–32.33]	27.59 [26.52–28.96]	< 0.05
LLC diagnosis and level of agreement	oLLC (W)	31/54, 57.4%	5/28, 17.9%	3/20, 15%	/
	nLLC (W)	36/54, 66.7%	17/28, 60.7%	7/20, 35%	/
	Cohen’s K	0.73 [0.543–0.912]	0.25 [0.035–0.459]	0.49 [0.111–0.876]	/
	McNemar Test	P = 0.125	P = 0.0005	P = 0.125	/
Biopsy	EMB (W)	16/42, 38.1%	24/42, 57.1%	2/42, 4.76%	< 0.05
	EMB diagnosis of AM (W)	6/16, 37.5%	22/24, 91.7%	2/2, 100%	< 0.05
	nTI (ms, median and CI) EMB +	1065 [1008–1121]	1095 [1074–1136]	/	< 0.05
	T2 (ms, median and CI) EMB +	53.33 [49.87–56.35]	51.88 [50.79–55.07]	/	< 0.05
	ECV (%, median and CI) EMB +	31.14 [26.05–36.15]	31.53 [27.92–34.18]	/	< 0.05
Diagnostic accuracy	oLLC	75%	41.7%	/	< 0.05
	nLLC	75%	66.7%	/	< 0.05

*IL* infarct-like, *CM* cardiomyopathic, *AR* arrhythmic, *CMR* cardiac magnetic resonance, *EDV* end diastolic volume, *BSA* body surface area, *CI* confidence interval, *EF* ejection fraction, *LLC* Lake Louise Criteria, *EMB* endomyocardial biopsy

### Comparison between oLLC vs nLLC

Inter-observer agreement was excellent for both oLLC and nLLC, resulting in a Cohen’s k of 0.94 and 0.98, respectively. In detail, the agreement was substantial for EGE ratio (k = 0.66) and excellent for LGE and edema assessment with T2w STIR (k was 0.94 and 0.90 respectively). As regards relaxometric images analysis, the agreement was almost perfect either for native T1 and for T2 mapping and for ECV (k was 0.96 and 0.96 and 0.89, respectively). Doubtful cases were discussed until an agreement between the two operators was found.

OLLC were able to diagnose acute myocarditis in 39 patients (39/102, 38.2%), vs 60 in nLLC (60/102, 58.8%), k = 0.57 [0.428–0.713], MNT p < 0.01.

**Fig. 1:**
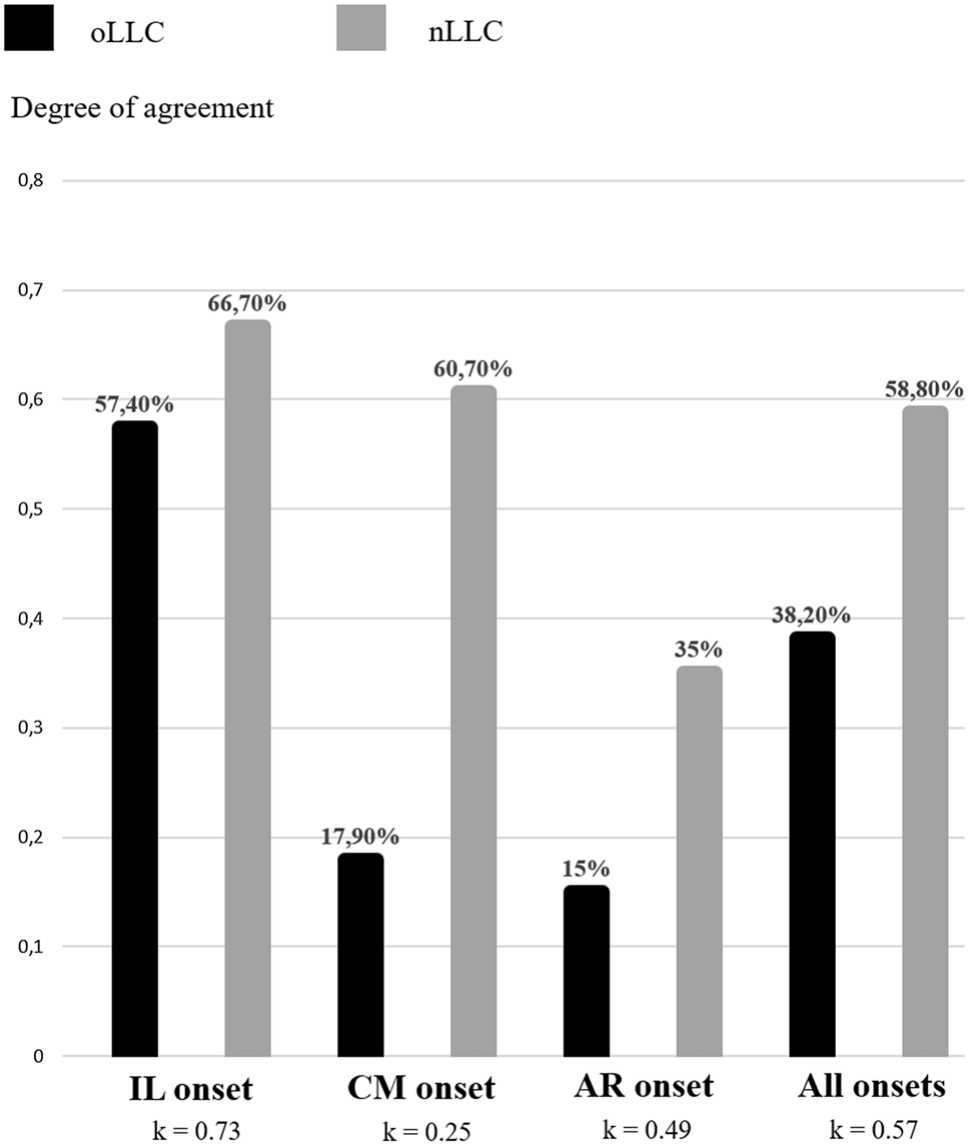
The degree of agreement between old and new LLC was good for the IL presentation (57.4% vs 66.7%, respectively) and fair for CM and AR onsets (17.9% vs 60.7% and 15% vs 35%, respectively). Cohen’s k was 0.57 considering the overall clinical patterns (38.2% vs 58.8%). *LLC* Lake Louise Criteria, *IL* infarct-like, *CM* cardiomyopathic, *AR* arrhythmic

In particular, diagnosis of CM onset was detected in 5 patients (5/28, 17.9%) according to oLLC and in 17 (17/28, 60.7%) with the nLLC (k = 0.25 [0.035–0.459], MNT p < 0.01). No statistical difference was found for AR onset, where oLLC were positive in 3 patients (3/20, 15%) and nLLC in seven of them (7/20, 35%), k = 0.49 [0.111–0.876], MNT p > 0.05 and for IL onset (oLLC 31/54, 57.4% vs nLLC 36/54, 66.7%, k = 0.73 [0.543–0.912], MNT p > 0.05, Fig. 1).

Mapping sequences were able to diagnose acute myocarditis when oLLC were negative in 5 patients for IL (5/54, 9.26%), 12 for CM (12/28, 42.8%) and 4 for AR presentation (4/20, 20%) (see Table 1).

Diagnostic performances of oLLC vs nLLC was also compared in a subcohort of 42 individuals with EMB.

Histological confirmation was available in 2 patients with AR onset (2/42, 4.76%), 24 with CM presentation (24/42, 57.1%) and 16 with IL pattern (16/42, 38.1%). According to EMB, acute myocarditis was present in 2 patients with AR onset (2/2, 100%), 22 patients with CM (22/24, 91.7%) and 6 patients with IL (6/16, 37.5%).

Given the limited number of AR individuals, analysis was carried out by only comparing CM and IL presentation.

**Fig. 2:**
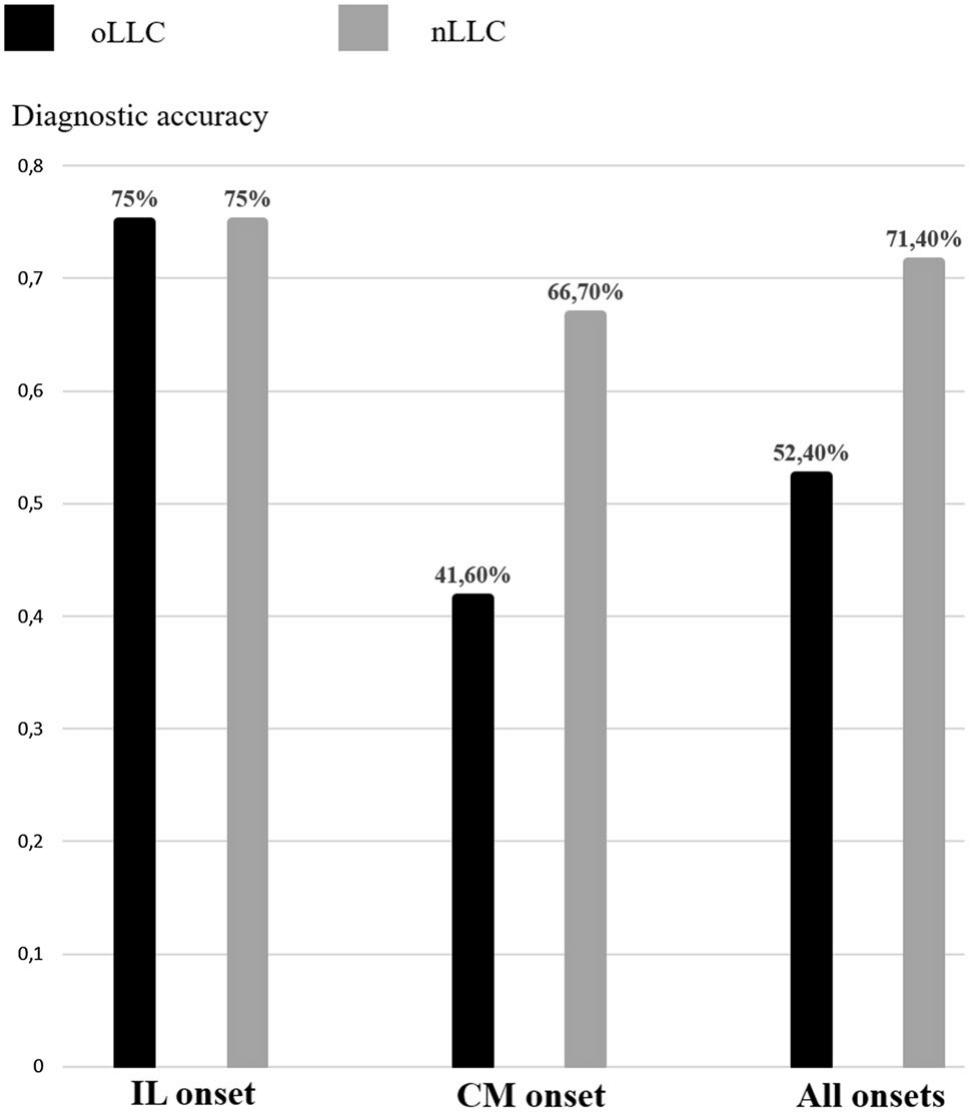
Comparison between old and new LLC as regards the diagnostic accuracy. DA was the same for oLLC and nLLC in IL onset. A great increase in DA of nLLC for CM onset (41.60% of oLLC vs 66.70% of nLLC) was found. The overall DA for oLLC was 52.40% vs 71.40% for the new criteria. *LLC* Lake Louise Criteria, *DA* diagnostic accuracy, *CM* cardiomyopathic

Diagnostic Accuracy (DA) in IL onset was 75% [47.62%–92.73%] for both oLLC and nLLC; in CM presentation, DA was 41.7% [15.17%–72.33%] with oLLC and 66.7% [34.89%–90.08%] with nLLC. DA for all clinical patterns (IL, CM and AR) was 52.3% [29.78%–74.29%] with oLLC and 71.4% [47.82%–88.72%] with nLLC (Table 1, Fig. 2). The NRI and NARI were 0.13 and 0.00 for IL, − 0.64 and 0.25 for CM and 0.07 and 0.19 for all clinical presentations, respectively.

## Discussion

### Infarct-like presentations

Intra-myocardial accumulation of water, together with endothelial dysfunction, represent the hallmarks of active inflammation which are usually found in IL forms of myocarditis [25] [26]. Corresponding CMR features are characterized by a typical non-ischemic distribution of myocardial damage consistent with tissue oedema and necrosis [6, 27]. This was confirmed in our patient’s cohort, where the vast majority of patients had a local, typically mid-basal lateral left ventricular involvement associated with preserved or slightly reduced ejection fraction, [6] (mean EF was 56.04% [54.38–58.87%] in IL group).

**Fig. 3:**
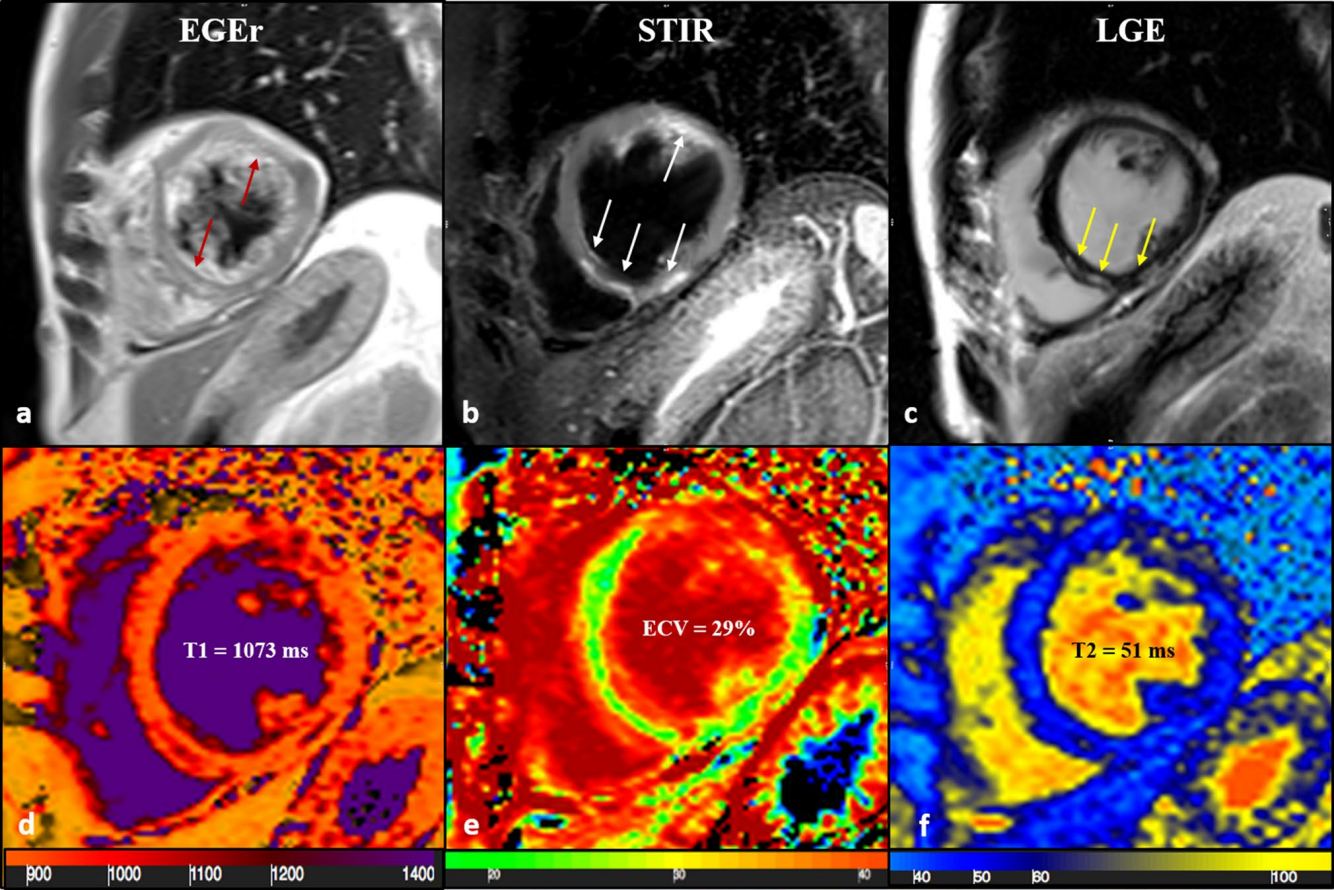
43-year-old male patient with acute chest pain and T-Troponine release (0.05 mcg/L). The EGE ratio (**a**) was 5.4 with hyperemia of the anterior and infero-septal segments on mid-ventricular plane (**a**, red arrows). Oedema (**b**) with a patchy distribution on the antero-lateral and infero-septal segments (**b**, white arrows), was confirmed by a T2 ratio of 2.4 on mid-ventricular plane. A non-ischemic LGE (**c**) was found in the inferior and infero-septal segments (**c**, yellow arrows) with a subepicardial distribution. Native T1 (**d**, mean value of 1073 ms), ECV (**e**, mean value of 29%) and T2 mapping (**f**, mean value of 51 ms) were all increased, confirming the positivity of both old and new LLC. *EGE* early gadolinium enhancement, *STIR* short tau inversion recovery, *LGE* late gadolinium enhancement, *ECV* extracellular volume fraction, *LLC* Lake Louise Criteria

In this specific setting, use of conventional imaging (T2w-STIR, PSIR-LGE and T1-TSE) provides sufficient information to display typical inflammation features like oedema [28], necrosis/fibrosis and hyperaemia, providing a visual and a semi-quantitative assessment of myocardial injury (Fig. 3).

As a consequence, we found no added value from the use of relaxometry-based sequences which yielded to comparable results between oLLC vs nLLC, for diagnostic performances with a substantial degree of agreement [0.73 (0.543–0.912)] and a DA of 75% [47.62%–92.73%] for both Criteria using EMB as a reference standard.

We also observed three cases of diffuse myocardial damage who were clinically categorized as fulminant myocarditis (FM). FM has been described as an acute disease due to a severe inflammatory response that requires mechanical or electrical support and the eventual management of threatening arrhythmias. Although a typical pattern of CMR myocardial damage could not be identified in this subgroup, oLLC well performed in those patients, showing an equal diagnostic performance as compared to nLLC. Our results are supported by the literature [29]. In fact, myocardial oedema, evaluated by T2w STIR, is usually well represented and involves different myocardial segments, with an elevated SI as compared to not injured myocardium [29], whereas LGE is associated with a worsening of cardiac function and development of heart failure after myocarditis [30, 31].

### Atypical forms: cardiomyopathic and arrhythmic presentations

CMR appearance of cardiomyopathic forms reflects the different evolution of myocardial damage which has been described as the result of a prolonged subacute inflammatory response [6] and myocytes apoptosis [21].

**Fig. 4:**
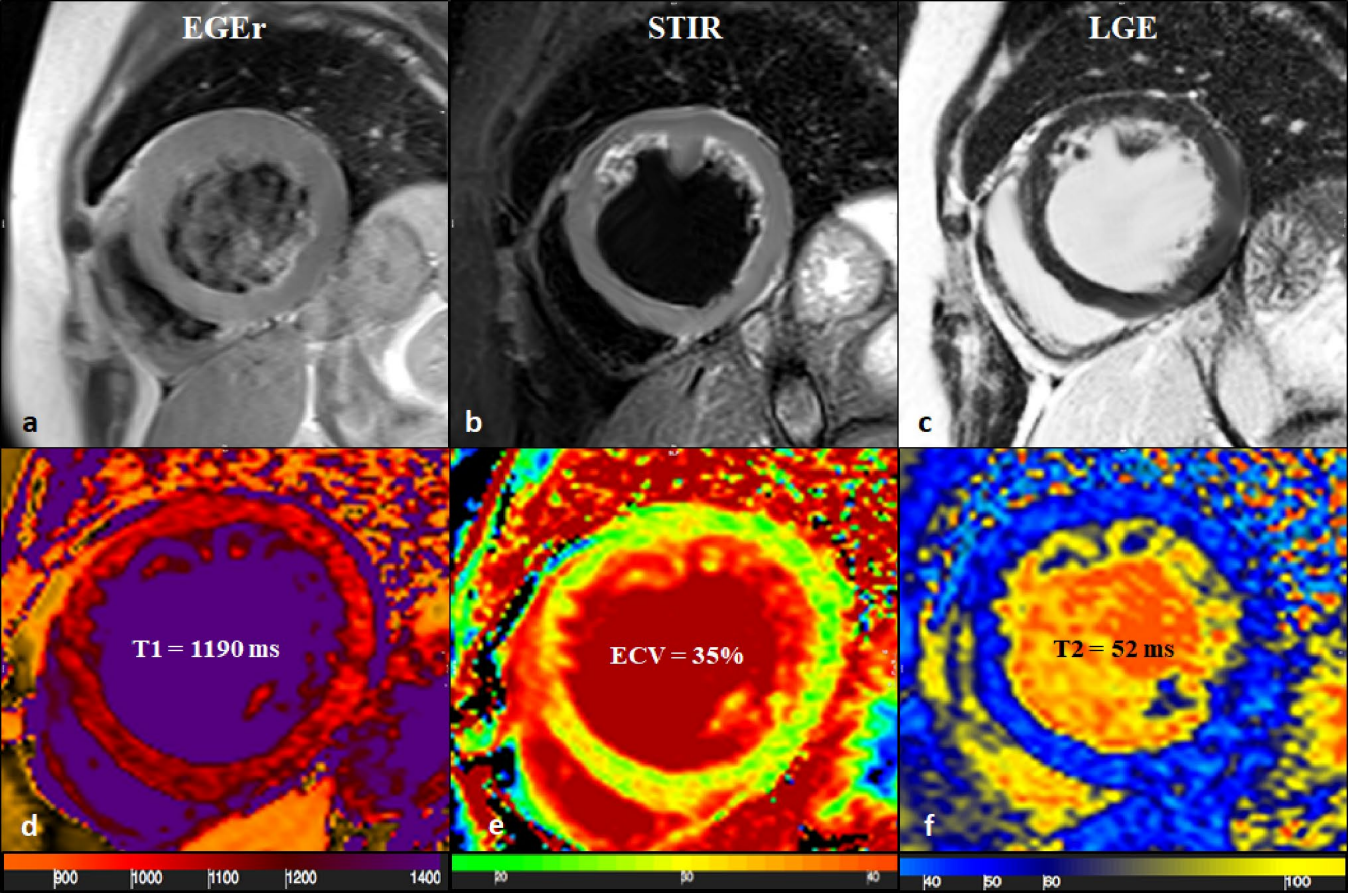
65-years old female with reduced EF (24%), LV dilation (EDV/BSA: 153 ml/mq) and no significant coronary artery disease. No hyperaemia (**a**, EGE ratio < 4), neither oedema (**b**, T2 ratio < 2) nor LGE (**c**) was found: oLLC were negative for suspected acute myocarditis. Native T1 was severely increased (**d**) with a mid-ventricular mean value of 1190 ms, ECV (**e**) mean value was 35% and T2 mapping was slightly increased (**f**, mean value: 52 ms). Both T1 and T2 criterion were positive according to nLLC and the diagnosis of viral myocarditis was confirmed by EMB. *EF* ejection fraction, *EDV* end diastolic volume, *BSA* body surface area, *EGE* early gadolinium enhancement, *STIR* short tau inversion recovery, *LGE* late gadolinium enhancement, *oLLC* old Lake Louise Criteria, *ECV* extracellular volume, *nLLC* new Lake Louise Criteria, *EMB* endomyocardial biopsy

LV dysfunction and clinical symptoms are usually preceded by viral persistence in myocardial tissue, which triggers the activation of autoimmune processes inducing subtle and diffuse myocardial damage [32]. Accordingly, there is a progressive oedema reabsorption, which corresponds to mild and diffuse signal intensity abnormalities that are poorly detectable with conventional sequences [33]. In this specific clinical setting, tissue relaxometry-based imaging allows quantification of T1 and T2 relaxations changes which yields to superior diagnostic performances as compared to conventional imaging (Fig. 4). Myocardial mapping imaging has proven to recognize myocardial injury after 4–8 weeks from the viral infection [34]. This diagnostic advantage has been confirmed in our CM patient’s population, where the DA of nLLC was 66.7% [34.89%–90.08%] and 41.6% [15.17%–72.33%] for oLLC, with a fair degree of agreement [0.25 (0.035–0.459)].

A similar potential mechanism has been hypothesized in arrhythmic disease onset, which pathogenesis still needs to be clarified. In some cases, arrhythmias originates from re-entrant mechanisms due to foci of fibrosis and extracellular matrix expansion that occur in the chronic phase of the infection [35]. It has also been speculated that AR pattern could be associated to Human Herpes virus 6, a neurotropic virus which directly affects conduction system cells, generating arrhythmias [6]. But ventricular arrhythmias are also attributed to ion channel dysfunction in the acute phase and to the pro-arrhythmic effect of cytokines released in the chronic phase of viral infection [36]. These micro-molecular alterations [37] in the absence of typical inflammation features are hardly detected even by mapping sequences. In addition, CMR in patients with AR onset often reveals low diagnostic quality images and motion artefacts due to arrhythmias [21]. However, nLLC allowed the diagnosis of AM in 7/20 patients, whereas oLLC resulted positive in 3/20 [k = 0.49 (0.111–0.876)]. A diagnostic comparison was not feasible in this specific patient’s cohort, due to the limited number of EMBs performed (2/20).

### Study limitations

Main limitations of our study concerns the small sample size and, in particular, the low number of patients who underwent EMB. Moreover, biopsy was only performed in those subject whose CMR and clinical diagnosis was unclear: according to clinical guidelines EMB was used as a diagnostic tool only in patients with heart failure with or without LV dilation and/or new onset arrhythmias [16] (thus, mainly CM and AR groups) and this could have introduced a selection bias for the evaluation of DA of oLLC and nLLC, affecting mostly IL presentation group. Indeed, IL patients who underwent the invasive procedure had inadequate clinical response to therapy or persistently depressed LV systolic function and this brought to a variable delay in EMB procedure with possible attenuation of the typical histopathological manifestations of myocarditis.

## Conclusions

Our study has shown a significant added diagnostic value of nLLC in patients with CM forms of myocarditis, reflecting the superior detection of subtle/diffuse forms of myocardial involvement characterizing myocardial mapping imaging. Conventional sequences remain highly accurate in IL forms, as a consequence of the predominant active inflammatory manifestations associated with acute symptoms with troponin raise and typical ECG-changes. No significant advantages were found in the application of nLLC in patients with AR onsets. Pathological substrate triggering arrhythmias in myocarditis remains debated, but likely depends on a combination of direct viral infection and/or structural damage to the conduction system which is poorly depictable with CMR imaging. As an additional peculiarity, image quality is highly influenced by the presence of arrhythmias, further limiting accuracy of the method in this specific clinical setting.

Larger prospective studies, also investigating on other imaging parameters such as myocardial strain, intra-cavitary fluid analysis or early myocardial T1 mapping [38], are expected to further clarify the role of CMR in different forms of myocarditis with the aim of tailoring specific scanning protocols to patient’s clinical presentations.

## Abbrevations

AM: Acute Myocarditis
AR: Arrhythmic
BSA: Body Surface Area
CI: Confidence Interval
CM: Cardiomyopathic
CMR: Cardiac Magnetic Resonance
DA: Diagnostic Accuracy
ECG: Electrocardiogram
ECV: Extracellular Volume
EDV: End Diastolic Volume
EF: Ejection Fraction
EGE: Early Gadolinium Enhancement
EMB: Endomyocardial Biopsy
FM: Fulminant Myocarditis
IL: Infarct-Like
GRE: Gradient Echo
LLC: Lake Louise Criteria
LGE: Late Gadolinium Enhancement
LV: Left ventricle
MOLLI: Modified Look Locker Inversion Recovery
oLLC: Old Lake Louise Criteria
nLLC: New Lake Louise Criteria
PCR: Polymerase Chain Reaction
PSIR: Phase Sensitive Inversion Recovery
ROI: Region of Interest
TSE: Turbo Spin Echo
